# Probiotics and Oral Complications of Antineoplastic Therapy in Head and Neck Cancer: A Systematic Review and Meta-Analysis

**DOI:** 10.3390/dj13060254

**Published:** 2025-06-06

**Authors:** Tanya Pereira-Riveros, Enric Jané-Salas, José López-López, Constanza Saka-Herran, Isabel Linares-Galiana, Teresa Vinuesa-Aumedes

**Affiliations:** 1 Department of Odontostomatology, Faculty of Medicine and Health Sciences (Dentistry), University of Barcelona, 08907 L’ Hospitalet de Llobregat, Barcelona, Spain; tanya.pereira@ub.edu (T.P.-R.);; 2 Microbiology Unit, Department of Pathology and Experimental Therapeutics, School of Medicine and Health Sciences, IDIBELL, University of Barcelona, 08907 L’ Hospitalet de Llobregat, Barcelona, Spain; 3 Head Service of the Medical-Surgical Area, Dentistry Hospital, Faculty of Medicine and Health Sciences (Dentistry), Bellvitge Campus, University of Barcelona, 08907 L’ Hospitalet de Llobregat, Barcelona, Spain; 4 Radiation Oncology Department, Catalan Institute of Oncology, 08907 L’Hospitalet de Llobregat, Barcelona, Spain

**Keywords:** radiotherapy, chemotherapy, surgery, probiotics, head and neck cancer

## Abstract

**Background/Objectives:** Patients with head and neck cancer frequently develop oral complications such as oral mucositis, infections, necrosis, and periodontal disease among others as a consequence of antineoplastic therapy. It is mainly radiotherapy that promotes oral dysbiosis, favouring the overgrowth of opportunistic microorganisms. Identifying effective adjunctive strategies to prevent or mitigate these adverse effects is crucial. Recent studies have suggested that probiotics could be used to restore microbial homeostasis and modulate inflammatory responses in the oral cavity. This systematic review and meta-analysis assessed the efficacy of probiotics in alleviating oral complications associated with antineoplastic treatments in this patient population. **Methods:** A comprehensive search was conducted in PubMed, LILACS, Scopus and the Cochrane Central Register of Controlled Trials, following the PRISMA 2020 guidelines. Only randomised controlled trials (RCTs) were included. **Results:** Nine eligible RCTs were analysed using a random-effects meta-analysis. Probiotic use was significantly associated with a reduced incidence of severe (grade 3–4) oral mucositis (RR = 0.58; 95% CI: 0.41–0.81). Moderate benefits were also observed in modulating the oral microbiota and reducing levels of pathogenic bacteria and *Candida* spp. However, no significant improvements were noted in periodontal parameters or plaque indices. **Conclusions:** Probiotics show promise in the management of oral mucositis, but further well-designed trials are needed to evaluate their broader impact on oral health during cancer therapy. This review is not registered on PROSPERO.

## 1. Introduction

Head and neck cancer (HNC) treatment involves multimodal strategies, including surgery, radiotherapy (RT), chemotherapy (CT), and targeted biological therapies [1,2,3]. The choice of treatment depends on tumour location, staging, and resectability [4]. These therapeutic modalities frequently lead to oral complications such as oral mucositis (OM), dysgeusia, salivary gland dysfunction, infections, necrosis, and periodontal disease, which significantly impact patients’ quality of life [5,6].

Radiotherapy, in particular, can alter the oral microbiota and promote dysbiosis, either through the direct cytotoxic effects of ionising radiation or indirectly via salivary gland damage, leading to reduced salivary flow, pH shifts, and nutrient imbalance [7]. These conditions favour the overgrowth of opportunistic microorganisms such as *Candida* spp., *Staphylococcus* spp., *Enterococcus faecalis*, and enteric bacilli [8]. This microbial imbalance may increase the susceptibility of the oral mucosa to inflammatory and infectious processes [9]. In contrast, surgical intervention may cause transient disturbances in the oral microbiota without leading to pronounced dysbiosis. For example, Kageyama et al. [10] observed shifts in microbial composition following tumour resection, but these changes did not result in the overgrowth of non-indigenous pathogenic species, indicating that surgery alone may not significantly disrupt microbial homeostasis. In this context, recent studies have proposed the use of probiotics as a potential strategy to restore microbial homeostasis and modulate inflammatory responses in the oral cavity [11,12,13]. Probiotics are defined as “live microorganisms that, when administered in adequate amounts, confer a health benefit to the host” [14]. Their effects in humans have been extensively studied, leading to multiple health, preventive, and therapeutic indications, particularly as effective agents for a range of gastrointestinal tract (GIT) disorders [15]. In the oral cavity, probiotics have been associated with a reduction in colony-forming unit (CFU) counts of cariogenic pathogens and the inhibition of periodontal pathogens. Additionally, they are believed to modulate the inflammatory response—both humoral and cellular—by producing substances such as lactic acid, hydrogen peroxide, and bacteriocins [16,17]. Previous systematic reviews, including that by Yang et al. (2024) [18], have primarily explored the role of probiotics in preventing oral mucositis among patients undergoing cancer therapy. While these investigations have provided valuable insights, other clinically significant complications, such as candidiasis, periodontal disease, and shifts in the oral microbiota, remain insufficiently explored in the current literature.

Given these gaps, and the potential of probiotics to counteract microbial shifts and inflammation, a further synthesis of the clinical evidence is warranted. Therefore, this systematic review and meta-analysis aims to evaluate the effects of probiotics on oral complications induced by antineoplastic therapies in patients with head and neck cancer. Specifically, it addresses the following research question:

Is the use of probiotics, compared to placebo, effective in reducing oral complications induced by radiotherapy, chemotherapy, or surgery in patients with head and neck cancer?

## 2. Materials and Methods

This systematic review was conducted in accordance with the Preferred Reporting Items for Systematic Reviews and Meta-Analyses (PRISMA) 2020 guidelines.

### 2.1. Eligibility Criteria

Studies were included in which the intervention involved the use of probiotics, compared with a placebo (or no intervention), for the treatment of oral complications induced by therapies for head and neck cancer. Only randomised clinical trials (RCTs) were considered eligible if they met the following criteria: (1) study participants were patients diagnosed with head and neck cancer; (2) patients had received chemotherapy, radiotherapy, chemoradiotherapy, and/or surgery as part of their antineoplastic treatment; and (3) the intervention group received probiotic agents or probiotic-containing products. The control group received a placebo, other therapeutic agents, or no treatment. Case reports, conference abstracts, non-randomised studies, and review articles were excluded. Only articles published between 2009 and 2024, in English or Spanish, were included in the review.

### 2.2. Sources of Information

An electronic search was conducted in four databases: PubMed, LILACS, Cochrane Central Register. andScopus. References of studies selected for inclusion were also searched. The most recent search was conducted on 1 September.

### 2.3. Search Strategy

An initial search strategy was developed for PubMed using both Medical Subject Headings (MeSH) and free-text terms: ((“Mouth Neoplasms” [MeSH] OR “Head and Neck Neoplasms” [MeSH]) OR (“oral cancer” [TIAB] OR “head and neck cancer” [TIAB])) AND (“Probiotics” [MeSH] OR probiotics [TIAB]), with a date restriction from 1 January 2009 to 1 September 2024 [PDAT]. As this strategy yielded few eligible studies, a second, broader search was conducted using the free-text terms “oral cancer” AND “probiotics”, applying the same date range. Equivalent search strategies were adapted and applied to all selected databases (PubMed, LILACS, Cochrane CENTRAL, and Scopus), respecting the syntax and indexing terms of each platform.

### 2.4. Selection of Studies

Initially, two authors (TPR and EJS) independently screened the titles and abstracts of all references retrieved from the databases. Studies that appeared to meet the inclusion criteria were selected for full-text review. Subsequently, two additional authors (TVA and CSH) screened the reference lists of these studies to identify other potentially eligible articles. In the second phase, TPR and EJS independently reviewed the full texts of all selected articles, excluding those that did not meet the predefined inclusion criteria. Discrepancies between reviewers were resolved by consensus among all six authors.

### 2.5. Data Collection Process and Elements

One author, JLL, extracted the data of interest from the studies included in the final selection: study, date, methods, participants, type of treatment, intervention, and results. A second author, ILG, verified the extracted data. Disagreements were resolved through discussion with a third author, TPR, who made the final decision in cases of disagreement.

### 2.6. Outcomes

The primary outcomes extracted included the incidence of oral mucositis and candidiasis, periodontal index scores, and O’Leary plaque index scores. Secondary outcomes encompassed changes in the oral and gut microbiota.

Additional data collected comprised participant demographics (age, sex), cancer type and stage, anticancer therapy details, probiotic intervention characteristics (strain, dose, administration route, duration), control group type, and study design.

### 2.7. Risk of Bias in Individual Studies

To assess any potential risk of bias, the authors critically appraised each study using the Cochrane Collaboration’s tool for assessing the risk of bias in randomized trials [19].

### 2.8. Effect Measures

For dichotomous outcomes, such as the incidence of oral mucositis and candidiasis, risk ratios (RRs) with corresponding 95% confidence intervals were calculated. For continuous outcomes, such as periodontal index scores and O’Leary plaque index scores, mean differences (MDs) were used to compare groups. Summary measures included percentages for categorical variables and mean differences for continuous variables. A *p*-value of <0.05 was considered statistically significant.

### 2.9. Summary of Results

A meta-analysis was performed using a random-effects model to account for variability between the included studies. Heterogeneity was assessed using the I^2^ statistic and was considered significant when the *p*-value was less than 0.1. The statistical significance of treatment effects was determined using a threshold of *p* < 0.05. The results were pooled and analysed using RevMan version 5.3 software. Due to the limited number of included studies and variability across interventions and outcomes, no subgroup or sensitivity analyses were performed.

### 2.10. Reporting Bias Assessment

To assess the risk of bias due to missing results in the synthesis, funnel plots were planned for the visual inspection of asymmetry when at least 10 studies were available per outcome. However, due to the limited number of included studies, this analysis could not be performed. Selective reporting bias in individual studies was assessed using the Cochrane Risk of Bias tool, which includes a specific domain for this type of bias.

### 2.11. Certainty Assessment

The certainty of the evidence for the main outcomes was not formally assessed using a structured tool such as GRADE, due to the limited number of included studies and the heterogeneity observed in the study designs and reported outcomes. Consequently, the conclusions were based on a critical appraisal considering the risk of bias, consistency of findings, and the direct applicability of the available evidence.

## 3. Results

### 3.1. Selection of Studies

**Figure 1:**
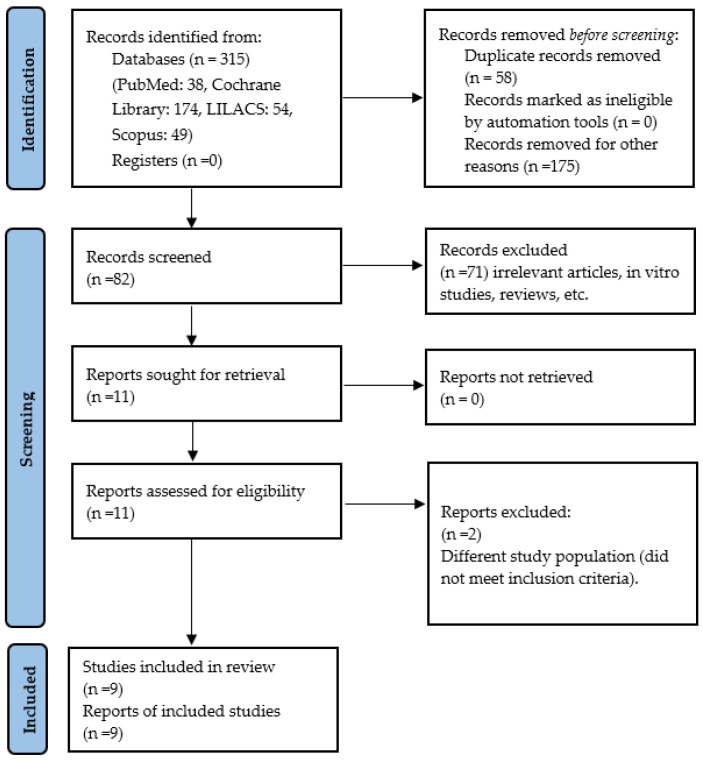
Flow chart of study selection.

A total of 315 references were identified across the selected databases: 38 from PubMed, 174 from the Cochrane Library, 54 from LILACS, and 49 from Scopus. After removing 58 duplicates and 175 records for other reasons, 82 records were screened based on titles and abstracts. Of these, 71 were excluded due to being irrelevant, in vitro studies, or review articles. The full texts of 11 articles were reviewed, with 2 excluded for not meeting the inclusion criteria due to involving a different study population [20,21]. Ultimately, nine studies were included in the qualitative synthesis and seven in the quantitative synthesis. Figure 1 shows the flowchart that was used to select the studies. Of these, seven articles assessed the effects of probiotics on oral mucositis [22,23,24,25,26,27,28], one evaluated the use of probiotics in oral candidiasis [29], and one examined the effects of probiotics on periodontal disease and plaque index [30]. In addition, one study analysed plaque and saliva samples using high-throughput sequencing [30], two studies assessed stool samples by high-throughput sequencing [23,28], and one study measured oral Candida counts by culture [29].

### 3.2. Characteristics of the Studies

Table 1 shows the main characteristics of the studies included in the review.

### 3.3. Risk of Bias in Individual Studies

**Figure 2:**
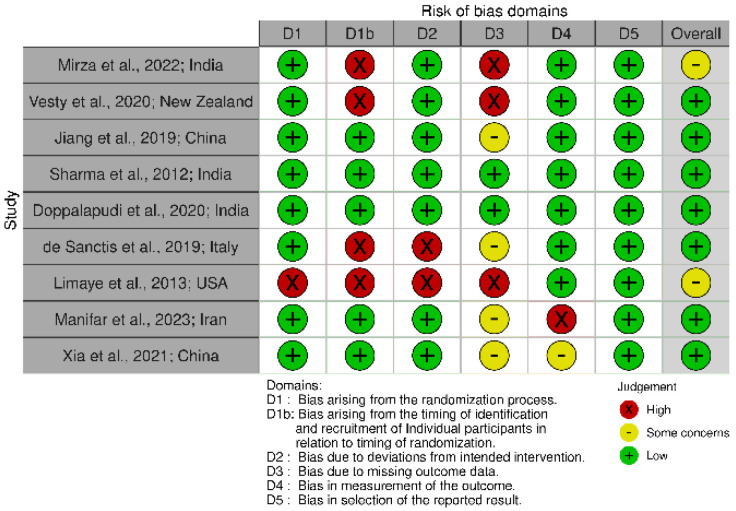
Risk of bias of individual studies [22,23,24,25,26,27,28,29,30].

The risk of bias among the studies is presented in Figure 2 [31]. The studies analysed varied in their methodological quality. Eight studies were assessed as having a low risk of bias in the domain of random sequence generation [22,23,25,26,27,28,29,30], while the study by Limaye et al. [24] was rated as having a high risk of bias. Regarding the allocation concealment domain, five studies were classified as low risk of bias [23,25,27,28,29] and four were classified as having a high risk of bias [22,24,26,30]. The blinding of participants and researchers was assessed as presenting a low risk of bias in seven studies [23,25,26,27,28,29,30]. Two studies were also assessed as presenting a low risk of bias in the blinding of outcome assessment domain [25,29], while in four other studies this domain was considered unclear [22,23,27,28]. Eight studies were assessed as presenting a low risk of bias regarding incomplete outcome data [22,23,24,25,26,28,29,30], and the selective reporting domain was assessed as presenting a low risk of bias in all included studies. Overall, the quality of the studies was rated as follows: two studies were considered to have a low risk of bias [25,29], two an unclear risk of bias [23,28], and five a high risk of bias [22,24,26,27,30].

### 3.4. Synthesis of Results by Outcomes of Interest

The summary data of the individual studies by outcomes of interest are presented in Table 2.

#### 3.4.1. Effects of Probiotics on Oral Mucositis (OM)

Data on the effects of probiotics on oral mucositis in head and neck cancer patients were reported in seven studies. Jiang et al. (2019) demonstrated that a probiotic combination significantly reduced the incidence of grade 2–3 OM compared to the placebo (*p* = 0.0001), although grade 1 OM worsened [23]. Sharma et al. (2012) reported a reduction in grade 3–4 OM and a significantly higher treatment completion rate in the *L. brevis* CD2 group versus the placebo (*p* = 0.001) [25]. Limaye et al. (2013) observed a 35% reduction in OM duration in the probiotic group, while all placebo recipients experienced ≥2 days of OM, and 29% of probiotic users reported OM for 0–1 day [24].

De Sanctis et al. (2019) found no significant difference in grade 3–4 oropharyngeal mucositis between groups (40.6% vs. 41.6%, *p* = 0.974) [22]. In contrast, Mirza et al. (2022) reported a significant reduction in high-grade OM (grade ≥ 3) in the probiotic group (*p* < 0.05) [26]. Manifar et al. (2023) showed a significant reduction in OM severity from the seventh session onwards, persisting throughout treatment. Severe OM (grade 3) occurred in 11.6% of the probiotic group versus 36.5% in the placebo group (*p* < 0.001) [27]. Xia et al. (2021) reported a significantly lower incidence of OM across all grades in the probiotic group (*p* < 0.01) [28].

**Figure 3:**
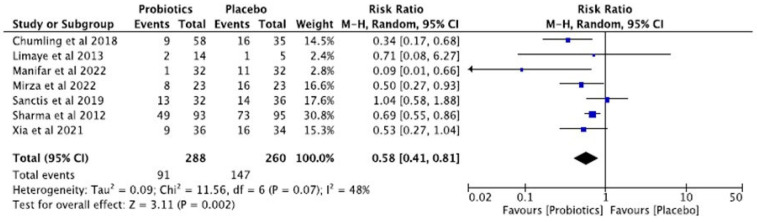
Forest plot of the pooled analysis comparing the incidence of grade 3–4 oral mucositis between probiotic and placebo groups [22,23,24,25,26,27,28]. Each individual study is represented by a square (relative risk) and its 95% confidence interval. The size of the square reflects the weight of the study. The black diamond represents the overall combined effect (RR = 0.58; 95% CI: 0.41–0.81), indicating a significant reduction in risk in the probiotic group.

Pooled analysis revealed that probiotic use was significantly associated with a lower incidence of grade 3–4 OM compared to the placebo (RR = 0.58; 95% CI: 0.41–0.81), corresponding to a relative risk reduction of approximately 42% (Figure 3). Moderate heterogeneity was observed (I^2^ = 48%, *p* = 0.07).

Although moderate heterogeneity was observed in the meta-analysis of oral mucositis outcomes (I^2^ = 48%), subgroup analyses were not conducted due to the limited number of studies available for each potential subgroup. The included studies differed considerably in key variables such as probiotic strain (e.g., *L. brevis* CD2, *S. salivarius* M18, symbiotics, recombinant strains), type of anticancer treatment (radiotherapy alone, chemoradiotherapy, or induction chemotherapy), and probiotic administration (form, dosage, and duration). Each subgroup would have comprised three or fewer studies, limiting statistical power and increasing the risk of spurious associations. Subgroup analysis was therefore deemed inappropriate.

A sensitivity analysis based on risk of bias was also considered. Of the nine studies reviewed, five were rated as having a high risk of bias in at least one key domain. Among the seven studies included in the meta-analysis of oral mucositis, four had a high risk of bias, two were rated as unclear, and only one was assessed as low risk. Excluding the high-risk studies would have reduced the dataset to just three studies, substantially limiting statistical power and compromising the reliability of the findings. Therefore, a formal sensitivity analysis was not conducted (Table 3).

#### 3.4.2. Effects of Probiotics on Oral Candidiasis

Doppalapudi et al. (2020) [29] evaluated the effects of probiotics, an antifungal agent, and a combination of both on oral counts of *Candida albicans* in patients with head and neck cancer undergoing radiotherapy. A statistically significant reduction in the colony-forming units (CFU/mL) of *Candida albicans* was observed in the three groups from baseline to post-intervention (*p* < 0.001).

Specifically, the probiotic group showed a decrease from 5339.2 ± 1194 to 400 ± 36 CFU/mL, while the antifungal group decreased from 4883.3 ± 1731 to 670 ± 52 CFU/mL. The combination group achieved the greatest reduction, from 5687 ± 1397 to 230 ± 42 CFU/mL [29].

#### 3.4.3. Effect of Probiotics on Plaque Index and Periodontal Screening

Vesty et al. (2020) reported improvements in the Community Periodontal Index of Treatment Needs (CPITN) in three patients: two out of six in the placebo group and one out of seven in the probiotic group. However, the difference between the groups was not statistically significant (*p* > 0.05). CPITN scores remained stable in all other participants. Regarding dental plaque, although the reduction was greater in the probiotic group, the difference remained non-significant (*p* > 0.05). The mean O’Leary plaque index decreased from 48% to 32% in the placebo group and from 37% to 26% in the probiotic group [30].

#### 3.4.4. Effect of Probiotics on Microbiota in Patients with Head and Neck Cancer

Vesty et al. (2020) [30] analysed bacterial plaque and saliva samples in patients with head and neck cancer using high-throughput sequencing to assess the effects of probiotic supplementation on microbial composition and diversity.

In terms of microbial composition, the most abundant genera in the plaque samples were *Prevotella intermedia* (17%), *Fusobacterium nucleatum* (15%), and *Veillonella* (12%). In saliva samples, the dominant genera were *Streptococcus* (35%), *Veillonella* (12%), and *Prevotella intermedia* (10%). With regard to alpha diversity, the number of observed species in plaque samples slightly declined in the probiotic group (from 176 ± 31 to 172 ± 21), while it increased in the placebo group (from 186 ± 17 to 196 ± 26). In saliva samples, species richness increased in both groups, from 177 ± 30 to 205 ± 25 in the probiotic group and from 165 ± 20 to 179 ± 23 in the placebo group. None of these changes reached statistical significance (*p* > 0.05) [30].

Jiang et al. (2019) reported that concurrent chemoradiotherapy (CCRT) disrupted intestinal microbial diversity in patients from the A-CCRT group (post-treatment with radiotherapy, chemotherapy, and placebo), whereas patients in the A-CCRT-P group (post-treatment with radiotherapy, chemotherapy, and probiotics) exhibited microbial diversity restored to levels comparable with healthy individuals (HP group) and with the B-CCRT-P group (baseline, prior to treatment) [23]. High-throughput sequencing analysis revealed that the predominant phyla across all groups were Firmicutes, Bacteroidetes, Proteobacteria, and Actinobacteria. Notably, Firmicutes were most abundant in the HP group (65.20%) and B-CCRT group (69.59%), but decreased significantly in the A-CCRT (51.62%) and A-CCRT-P (46.03%) groups. Bacteroidetes increased from 18.70% in B-CCRT to 30.45% in A-CCRT-P. A marked increase in Proteobacteria was observed in the A-CCRT-P group (17.12%) compared to the HP group (4.57%) and B-CCRT group (8.90%). Similarly, Actinobacteria showed a rising trend in the A-CCRT (9.36%) and A-CCRT-P (5.03%) groups versus the healthy baseline (1.18%).

These compositional shifts suggest that while probiotic supplementation may partially restore diversity, some dysbiosis persists post-treatment, particularly with elevated levels of Proteobacteria and Actinobacteria [23]. Xia et al. (2021) analysed intestinal microbiota composition using high-throughput sequencing. In the placebo group (ARCP), Firmicutes decreased to 52.10%, while Bacteroidetes and Actinobacteria increased to 27.22% and 8.18%, respectively, compared to healthy controls (66.03%, 28.02%, and 1.49%). In the probiotic group (ARCPM), Firmicutes rose to 63.30% and Actinobacteria declined to 3.00%, indicating a partial restoration of microbial balance. Proteobacteria remained elevated in both post-treatment groups (11.89% in ARCP and 11.13% in ARCPM) versus 4.45% in healthy controls [28].

## 4. Discussion

The available evidence is not yet sufficiently robust to support definitive conclusions; further research is needed to provide more solid results regarding the benefits of probiotics in managing oral complications associated with antineoplastic therapy in patients with head and neck cancer. To date, most studies have concentrated predominantly on oral mucositis. This review builds upon the findings of Yang et al. (2024) [18], broadening the scope to include additional complications such as periodontal disease, xerostomia, plaque accumulation, and alterations in the oral microbiota.

It is important to note that probiotic delivery was not consistent across the included studies, which may have contributed to the variability observed in the outcomes. The forms of administration included capsules, lozenges, mouth rinses, and symbiotic formulations, each differing in terms of bioavailability and duration of local action. Lozenges and rinses, by remaining in contact with the oral mucosa for longer, may favour more effective colonisation and modulation of the oral microbiota, in contrast to capsules, whose action is primarily gastrointestinal [32]. Moreover, colony-forming unit (CFU) counts varied substantially, which may also have influenced clinical efficacy. Thus, both the mode of administration and the probiotic dose should be regarded as potential confounding factors and important variables to standardise in future research.

Oral mucositis is a frequent adverse effect of radiotherapy and/or chemotherapy, associated with substantial morbidity. The risk increases significantly with concurrent chemoradiotherapy or cumulative radiation doses of 5000 cGy or more [33]. Moreover, the combination of chemotherapy and radiotherapy has been shown to increase the risk of grade 3 mucosal toxicity fourfold compared to radiotherapy alone [34]. Growing evidence also suggests a correlation between the dysbiosis of the oral microbiota and the severity of treatment-induced mucositis [35,36,37].

Despite various preventive strategies, few interventions have consistently demonstrated effectiveness in reducing mucositis. The mechanisms by which probiotics may alleviate this condition remain unclear. Nonetheless, preclinical studies, such as the one undertaken by Gupta et al. (2020), have shown that *Lactobacillus reuteri* DSM 17938 and PTA 5289 significantly reduce chemotherapy-induced mucosal damage in rodent models, primarily through a reduction in oxidative stress and inflammation [38].

Lin CW et al. (2022) reported that oral probiotic tablets enhanced immune function by increasing saliva production and salivary IgA levels, which may contribute to a reduction in oral pathogenic bacteria [12]. Similarly, Lin B et al. (2021) found that probiotics helped restore microbial diversity in both the oral cavity and gastrointestinal tract, while reducing the prevalence of species associated with xerostomia, including *Prevotella*, *Haemophilus*, *Fusobacterium*, and *Lautropia* [20].

### 4.1. Fungal Colonisation and Oral Candidiasis

Regarding fungal colonisation, a systematic review by Lalla et al. (2011) reported that the prevalence of oral fungal colonisation increased from 48.2% before cancer therapy to 72.2% during treatment, and remained at 70.1% following the completion of therapy [39]. This highlights the elevated risk of clinically significant oral fungal infections during and after antineoplastic treatment. While *Candida albicans* remains the most common aetiological agent of oral candidiasis, the incidence of resistant strains such as *C. krusei* and *C. dubliniensis* is increasing [40]. This growing resistance to antifungal agents underscores the urgent need for alternative therapies [41]. In this context, probiotics have shown promising potential. For example, Doppalapudi et al. (2020) demonstrated a reduction in oral Candida colony counts following probiotic administration in patients undergoing radiotherapy [29]. In vitro studies have further supported the antifungal properties of *L. reuteri*. Jørgensen et al. (2017) reported its inhibitory effects against *C. albicans*, *C. dubliniensis*, *C. tropicalis*, *C. parapsilosis*, and *C. glabrata*, although not *C. krusei* [42]. While some clinical trials have suggested reduced Candida colonisation with probiotic use, more targeted research is needed to determine their effectiveness in treating treatment-induced oral candidiasis [43,44,45].

### 4.2. Periodontal Health and Microbiological Balance

The complementary use of probiotics in periodontal therapy remains a matter of debate. While several studies have reported clinical, microbiological, and immunological benefits when probiotics are used alongside scaling and root planing [46,47,48,49,50], others have not found additional therapeutic effects [51,52]. Further high-quality research is required to clarify their role in periodontal management.

The clinical applicability of probiotics also deserves consideration. Most of the strains used in the included studies—such as *L. brevis* CD2, *S. salivarius* M18, and *B. longum*—are commercially available and have an established safety record. Reported adverse events were minimal; for example, Limaye et al. 2013 [24], noted only mild nausea in three participants. Although no serious complications were observed, caution is still warranted when administering probiotics to immunocompromised patients, and future studies should monitor for potential risks such as infection or microbial translocation.

While several mechanisms have been proposed to explain the benefits of probiotics—such as the modulation of the microbiota and attenuation of inflammation—only some of the included studies assessed these directly. Specifically, three studies (Vesty et al., Jiang et al., and Xia et al.) [23,28,30] evaluated changes in microbial composition using high-throughput sequencing, reporting shifts in bacterial diversity and relative abundance associated with probiotic use. Moreover, Jiang et al. and Xia et al. [23,28] also investigated immune parameters, showing significantly reduced rates of decline in CD3+, CD4+, and CD8+ T lymphocytes in patients receiving probiotic supplementation during chemoradiotherapy. These findings provide preliminary clinical evidence supporting the immunomodulatory and microbiota-stabilising effects of probiotics. Nonetheless, further trials incorporating both biological and clinical endpoints are needed to better clarify these underlying mechanisms.

### 4.3. Future Directions

To date, most research on probiotics in the context of head and neck cancer has focused on oral mucositis. However, future clinical trials should explore their potential as adjuvant or preventive treatments for other complications, including dysgeusia, xerostomia, altered salivary pH, periodontal disease, dental caries, and shifts in the oral microbiome. These conditions significantly affect patient quality of life and long-term wellbeing. Moreover, future studies should aim to define the optimal probiotic composition, dosage, and delivery method in order to maximise therapeutic benefit.

## 5. Conclusions

According to the reviewed literature, probiotics appear to exert a beneficial effect on oral mucositis in patients with head and neck cancer undergoing antineoplastic treatment, particularly in cases of grade 3 or 4 severity, both during and following therapy. However, further well-designed randomised clinical trials with a low risk of bias are required to evaluate their potential efficacy in the prevention or management of other oral complications commonly associated with antineoplastic therapies. The current evidence remains limited and does not yet permit definitive conclusions to be drawn.

## Figures and Tables

**Table 1:** The descriptive characteristics of the included studies.

Author, Year, Country	Study Design	Participants/Treatment	Probiotic Intervention	Evaluation/Outcome
de Sanctis et al., 2019, Italy [22]	Phase III RCT, open-label, multicentre	*n* = 68; age ≥ 18; 1:1 allocation; chemoradiotherapy or biological agent + RT	*L. brevis* CD2 (2 × 10^9^ CFU, 6 lozenges/day from day 1 of RT to 1 week after); control: sodium bicarbonate mouthwash	No benefit of *L. brevis* CD2 in reducing severe OM
Jiang et al., 2019, China [23]	RCT, double-blind, placebo-controlled	*n* = 99; age 18–70; 2:1 allocation; CCRT for nasopharyngeal carcinoma	*B. longum*, *L. lactis*, *E. faecium* (3 capsules/day during treatment); control: placebo	Significant reduction in OM incidence
Limaye et al., 2013, USA [24]	RCT, single-blind, placebo-controlled	*n* = 25; mean age 54; 5:2 allocation; induction chemotherapy	AG013 mouthwash (*L. lactis* recombinant); 1/3/6 times daily, days 1–14 of cycle 2 (induction QT)	35% decrease in OM days in probiotic group
Sharma et al., 2012, India [25]	RCT, double-blind, placebo-controlled	*n* = 200; mean age 50; 1:1 allocation; chemoradiotherapy	*L. brevis* CD2 (2 × 10^9^ CFU, 6 lozenges/day); from day 1 to 1-week post-treatment	Significant reduction in grade III–IV OM
Mirza et al., 2022, India [26]	RCT, double-blind, placebo-controlled	*n* = 46; age 30–60; 1:1 allocation; radiotherapy	*Bacillus clausii* (2 × 10^9^ spores, oral suspension, twice/day for 30 days); control: placebo	Grade ≥ 3 OM: 33% (probiotic) vs. 67% (placebo)
Doppalapudi et al., 2020, India [29]	RCT, 3-arm trial	*n* = 86; age 24–80; radiotherapy	*L. acidophilus*, *L. rhamnosus*, *B. longum*, *S. boulardii*; probiotic sachets 3×/day, 30 days; control: antifungal, probiotics and antifungals	Reduction in *C. albicans* in probiotic and combo groups
Vesty et al., 2020, New Zealand [30]	RCT, double-blind, placebo-controlled (pilot)	*n* = 13; age 40–70; 1:1 allocation; radiotherapy	*S. salivarius* M18 (3.5 × 10^9^ CFU, 1 lozenge/day for 4 weeks)	No significant change in microbiota; slight improvement in periodontal scores
Manifar et al., 2023, Iran [27]	RCT, double-blind, placebo-controlled	*n* = 64; age 20–70; 1:1 allocation; IMRT	Symbiotic mouthwash (*Bifidobacterium*, *Lactobacillus*, *Streptococcus* + prebiotic); 3×/day; day from 1 of RT to 1 week after	Significant reduction in OM severity
Xia et al., 2021, China [28]	Phase II RCT, placebo-controlled	*n* = 77; age 18–70; 1:1 allocation; CCRT	Modified strains: *L. plantarum*, *B. animalis*, *L. rhamnosus*, *L. acidophilus*; (1 capsule/day, during treatment)	Reduction in OM severity; improved immunity and microbiota balance

Abbreviations: RCT, randomized controlled trial; RT, radiotherapy; OM, oral mucositis; CCRT, concurrent chemoradiotherapy; CFU, colony-forming units; QT, chemotherapy; IMRT, intensity-modulated radiation therapy.

**Table 2:** Results of the individual studies by sections of interest.

Author, Year, Country	Evaluation Focus	Key Results
Jiang et al., 2019, China [23]	Effect of probiotics on oral mucositis	Significant reduction in grades 2–3 OM (*p* < 0.0001)
Sharma et al., 2012, India [25]	Effect of probiotics on oral mucositis	Reduced incidence of grade 3–4 OM (*p* = 0.001)
Limaye et al., 2013, USA [24]	Effect of probiotics on oral mucositis	35% reduction in OM days in probiotic group
de Sanctis et al., 2019, Italy [22]	Effect of probiotics on oral mucositis	No significant difference (*p* = 0.974)
Mirza et al., 2022, India [26]	Effect of probiotics on oral mucositis	Reduced grade ≥ 3 OM: 43% vs. 68%; *p* < 0.05
Manifar et al., 2023, Iran [27]	Effect of probiotics on oral mucositis	Significant reduction in grade 3 OM (*p* < 0.001)
Xia et al., 2021, China [28]	Effect of probiotics on oral mucositis	Lower OM severity across all grades (*p* < 0.01)
Doppalapudi et al., 2020, India [29]	Effect on oral candidiasis	Reduced *Candida* counts in probiotic and combination groups (*p* < 0.01)
Vesty et al., 2020, New Zealand [30]	Effect on periodontal status and microbiota	No significant change in plaque or CPITN; microbiota remained stable (*p* > 0.05)
Jiang et al., 2019, China [23]	Effect on gut microbiota	Probiotic group showed restored diversity, similar to healthy controls
Xia et al., 2021, China [28]	Effect on gut microbiota	Improved microbial balance post-probiotic intervention

**Table 3:** Summary of the number of studies per subgroup category.

Subgroup Category	Number of Studies	Comment
Radiotherapy only	4	Heterogeneous probiotic formulations
Chemoradiotherapy	3	Different treatment protocols and dosing schedules
Probiotics containing *L. brevis*	2	Differences in clinical context, treatment protocols, and timing of administration
High risk of bias	5	Issues with allocation concealment and reporting consistency
Low risk of bias	2	Consistent results; both included in the meta-analysis

## Data Availability

The data supporting the findings of this study are available from the corresponding author upon reasonable request.

## Protocol and Registration

This systematic review is not registered on any public database such as PROSPERO.

## Abbreviations

The following abbreviations are used in this manuscript:

RCT	Randomized controlled trial
RT	Radiotherapy
OM	Oral mucositis
CCRT	Concurrent chemoradiotherapy
CFU	Colony-forming units
QT	Chemotherapy
IMRT	Intensity-modulated radiation therapy
D	Day
CPITN	Community Periodontal Index of Treatment Needs
